# Accelerated multi-contrast high isotropic resolution 3D intracranial vessel wall MRI using a tailored k-space undersampling and partially parallel reconstruction strategy

**DOI:** 10.1007/s10334-018-0730-8

**Published:** 2019-01-03

**Authors:** Niranjan Balu, Zechen Zhou, Daniel S. Hippe, Thomas Hatsukami, Mahmud Mossa-Basha, Chun Yuan

**Affiliations:** 1 Vascular Imaging Lab, Department of Radiology, University of Washington, 850, Republican Street, Box 358050, Seattle, WA 98019 USA; 2 Healthcare Department, Philips Research China, Shanghai, China; 3 Department of Surgery, University of Washington, Seattle, USA

**Keywords:** Magnetic resonance imaging, Intracranial atherosclerosis, Cerebrovascular stroke

## Abstract

**Objective:**

To develop a 3D multi-contrast IVW protocol with 0.5-mm isotropic resolution and a scan time of 5 min per sequence.

**Materials and methods:**

Pre-contrast T1w VISTA, DANTE prepared PDw VISTA, SNAP, and post-contrast T1w VISTA were accelerated using cartesian undersampling with target ordering method (CUSTOM) and self-supporting tailored k-space estimation for parallel imaging reconstruction (STEP). CUSTOM + STEP IVW was compared to full-sample IVW, SENSE-accelerated IVW, and CUSTOM + zero-filled Fourier reconstruction in normal volunteers and subjects with intracranial atherosclerotic disease (ICAD). Image quality, vessel delineation, CSF suppression, and blood suppression were compared.

**Results:**

CUSTOM + STEP vessel wall delineation was comparable to full-sample IVW and better than SENSE IVW for vessel wall delineation on T1w VISTA and luminal contrast on SNAP. Average image quality and wall depiction were significantly improved using STEP reconstruction compared with zero-filled Fourier reconstruction, with no significant difference in CSF or blood suppression.

**Conclusions:**

CUSTOM + STEP allowed multi-contrast 3D 0.5-mm isotropic IVW within 30 min. Although some quantitative and qualitative scores for CUSTOM − STEP were lower than fully sampled IVW, CUSTOM + STEP provided comparable vessel wall delineation as full-sample IVW and was superior to SENSE. CUSTOM + STEP IVW was well tolerated by patients and showed good delineation of ICAD plaque.

## Background

Intracranial vessel wall MRI is an emerging imaging technique that is gaining traction in the characterization and differentiation of intracranial vasculopathies [1]. IVW has preliminarily shown the ability to differentiate etiologies of steno-occlusive disease of the carotid termini better than luminal imaging alone [2], characterize vulnerable aneurysm [3] and intracranial atherosclerosis [4], and distinguish between non-occlusive intracranial vasculopathies [5]. The major limitations of IVW, specifically long scan times and increased signal demand, arise secondary to the spatial resolution demands necessary for the evaluation of the small intracranial arteries with thin walls and complex vessel geometry, with the recommendation of using sequences with 0.5-mm isotropic voxel size [6]. Moreover, lesion characterization/differentiation may benefit from a multi-contrast IVW protocol which includes several sequences [5] that may further extend IVW protocol scan times, contributing to increased patient discomfort, degraded image quality due to patient motion, and limiting the feasibility of performing routine clinical IVW scanning. Accelerated IVW protocols that can adequately maintain high resolution and signal-to-noise ratio (SNR) have the potential to promote clinical translation of the technique for improved vascular disease assessment.

Imaging acceleration algorithms recently have been receiving increased attention due to the possibility of shortening scan times while maintaining image quality. Cartesian undersampling with target ordering method (CUSTOM) [7] and self-supporting tailored k-space estimation for parallel imaging reconstruction (STEP) [8] are recently developed k-space undersampling and partially parallel imaging reconstruction algorithms that have shown improved reconstruction accuracy and reduced noise amplification relative to the other existing partially parallel imaging techniques such as SPIRiT [9] and SAKE [10].

The goal of this study was, therefore, to develop an accelerated three-dimensional (3D) multi-contrast IVW protocol with 0.5-mm isotropic resolution with a scan time of 5 min per sequence using CUSTOM + STEP technology and to evaluate its performance as compared to fully sampled data acquisition and to other clinically available methods for reducing acquisition time. Images were acquired from both normal volunteers and two patients with confirmed intracranial atherosclerotic disease (ICAD) and assessed to answer the following questions: (1) whether adequate vessel wall delineation can be obtained when compared to full-sample acquisition, (2) whether CUSTOM + STEP performance is better than current clinically available parallel imaging acceleration, and (3) whether performance of STEP reconstruction is better than standard zero-filled image reconstruction clinically available on the scanner.

## Materials and methods

**Fig. 1:**
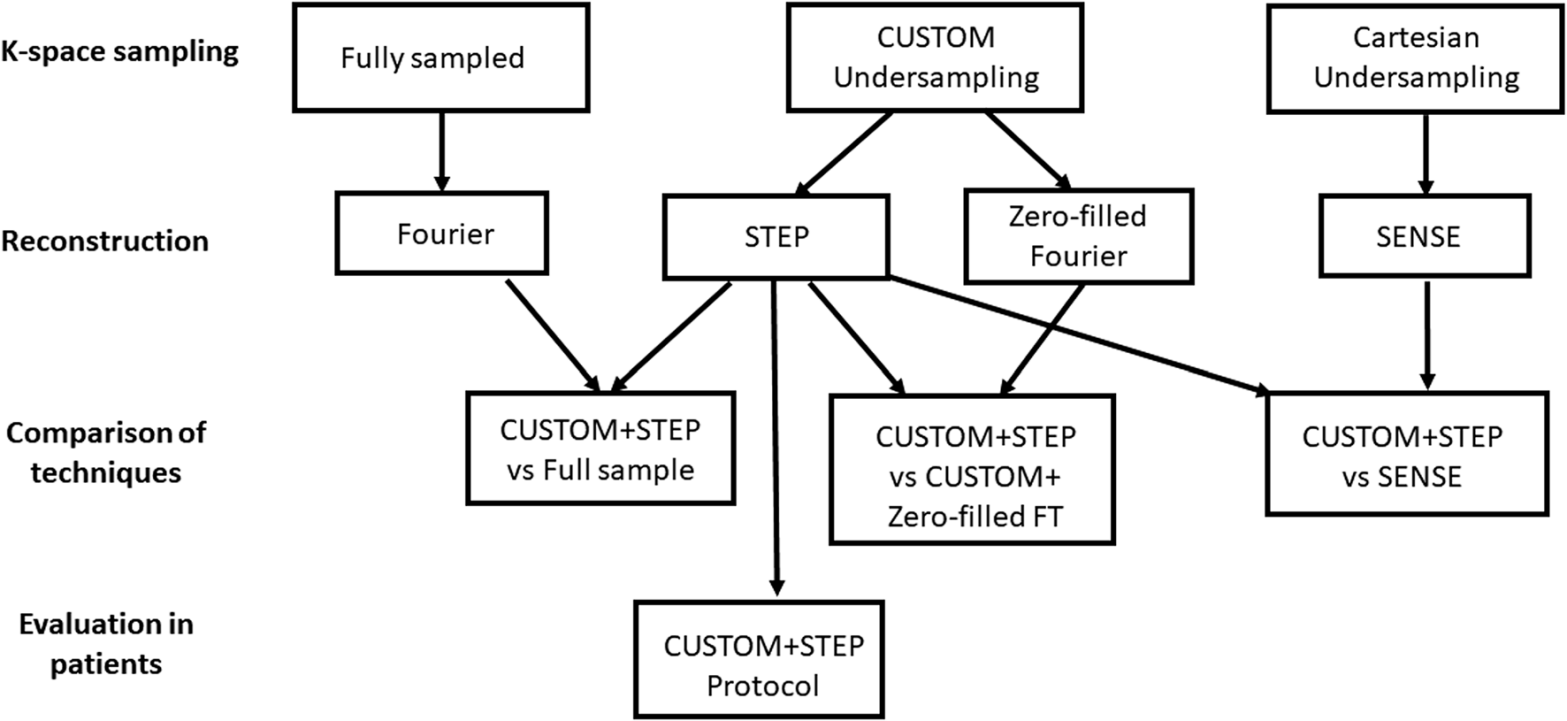
Flowchart showing MRI acquisition, reconstruction, and comparison steps. Scans with all three k-space sampling patterns (full sample, CUSTOM, and Cartesian undersampling) were acquired in volunteers and three comparisons (CUSTOM + STEP vs full sample, CUSTOM + STEP vs CUSTOM + Zero-fill Fourier transform, and CUSTOM + STEP vs SENSE) were performed in volunteers. Only the CUSTOM + STEP protocol was carried out in ICAD patients

All procedures performed in studies involving human participants were in accordance with the ethical standards of the institutional and/or national research committee and with the 1964 Helsinki declaration and its later amendments or comparable ethical standards. A flowchart of MR imaging, reconstruction, and comparison steps is shown in Fig. 1.

### MR imaging

Scans were carried out on a Philips Ingenia 3T (Philips Healthcare; Best, The Netherlands) scanner with 32-channel head coil. The target 3D intracranial wall imaging protocol (3D-WALLI) consists of four sequences (Table 1) all at 0.5-mm × 0.5-mm × 0.5-mm acquired resolution (interpolated to 0.25 mm × 0.25 mm × 0.25 mm). T1 weighting (T1w) and proton density weighting (PDw) were implemented using a variable flip angle Volume ISotropic Turbo spin echo Acquisition (VISTA) acquisition. PDw VISTA was acquired with Delay Alternating with Nutation for Tailored Excitation (DANTE) preparation [11] for CSF suppression [12]. Simultaneous Non-contrast Angiography and intraPlaque hemorrhage imaging (SNAP) [13] was acquired in two axial imaging slabs to maintain blood replacement requirements of the sequence and cover an 80-mm field-of-view in the head–foot direction. Post-contrast T1w VISTA was acquired with identical parameters to pre-contrast T1w VISTA. Scan times and imaging parameters for the accelerated 3D-WALLI protocol are provided in Table 1. To reduce scan times of 3D-WALLI sequences to a target of 5 min per sequence, k-space undersampling with CUSTOM [7] and reconstruction with STEP [8] were adopted. Post-contrast T1w-VISTA was scanned 3 min after single-dose gadolinium contrast injection (0.1 mmol/kg of Prohance at 2 cc/s) in patients with ICAD.

**Table 1:** Multi-contrast ICAD IVW Imaging Parameters

Sequence	T1w VISTA	PDw DANTE VISTA	Post T1w VISTA	SNAP^e^	3D-TOF
Orientation	Coronal^a^	Coronal^a^	Coronal^a^	Axial	Axial
Field-of-view	180 × 180 × 40	180 × 180 × 40	180 × 180 × 40	180 × 180 × 40	190 × 190 × 72
Resolution (mm)	0.5 × 0.5 × 0.5	0.5 × 0.5 × 0.5	0.5 × 0.5 × 0.5	0.5 × 0.5 × 0.5	0.5 × 0.5 × 1
TR (ms)	800	2000	800	11	15
TE (ms)	26	36	26	6.3	3.5
FA (˚)	Varies	Varies	Varies	11/5	18
Echo train length	30	60	30	98	NA
Averages	2	2	2	2	1
Bandwidth (Hz/pixel)	620	625	620	290	289
Preparation	No	DANTE^d^	No	Inversion	No
Fat suppression	Yes	Yes	Yes	Yes	No
Acc. rate^b^	4.5	4.5	4.5	4	2^c^
Scan time (min:s)	5:03	5:38	5:03	5:47^f^	5:15

^a^Axial orientation was obtained for CUSTOM + STEP vs full-sample and CUSTOM + STEP vs SENSE comparisons

^b^CUSTOM acceleration rate

^c^SENSE acceleration rate

^d^See Ref. [11]

^e^SNAP was acquired in two separate axial slabs with overlap of slices at slab margins

^f^Scan time for one SNAP slab

### k-Space undersampling with CUSTOM

Parameters for CUSTOM were selected based on required acceleration to achieve approximately a 5-min scan time per sequence. CUSTOM undersampling was previously developed to provide adequate support for both parallel imaging and compressed sensing reconstructions [7]. For 3D-WALLI protocol, an auto-calibration region of size 26 × 26 voxels was fully sampled. Given a targeted acceleration factor, CUSTOM undersampling pattern was generated point by point using a parametric variable radius sampling scheme. Optimized CUSTOM parameters σ_l_ and σ_g_ that correspond to the shape parameter of the local and global sampling distributions were set to 0.22 and 0.33 [7]. Acceleration factors of 4.5X were used for PDw VISTA, pre-contrast T1w-VISTA and post-contrast T1w-VISTA and 4X for SNAP to achieve a 5-min scan time for each sequence.

### STEP parallel image reconstruction

Scanner RAW format multichannel k-space data were exported to a separate workstation for offline processing. STEP reconstruction was carried out using Matlab (Mathworks Inc, Natick, USA) on a 64-bit Windows workstation fitted with a multi-core CPU and a GPU video-card. Workstation specifications are as follows: Intel Xeon Processor 2.4 GHZ eight cores, 32 GB RAM, 1 GB hard drive, and NVIDIA GeForce GTX TITAN X GPU with 3584 cores. The Matlab Parallel Computing Toolbox was employed to recruit all eight CPU cores for parallel processing. The two images from SNAP (inversion-recovery and reference) were jointly reconstructed and SNAP-corrected real images were calculated.

### Comparison to fully sampled protocol

Fully sampled and CUSTOM undersampled images were obtained in six volunteers (age 28–66 years, mean age 41 years, 1 male, and 5 female). No gadolinium contrast was administered. CUSTOM undersampled images were reconstructed using STEP. Imaging parameters for the fully sampled protocol were identical to the CUSTOM undersampled data set (given in Table 1) except for the fact that no acceleration rate was applied and a single average was acquired. Scan times for fully sampled 0.5-mm isotropic PDw VISTA, T1w VISTA and SNAP were 12:38, 10:04, and 12:24 min, respectively, with no averaging. Vessel wall delineation was compared on PDw VISTA and T1w VISTA. Since SNAP is not intended to show the vessel wall, lumen contrast was compared for SNAP. The CUSTOM undersampled images were STEP reconstructed offline and image quality was compared by a neuroradiologist as described below.

### Comparison to SENSE parallel imaging acceleration

Parallel imaging with SENSE is widely available on current scanners and is an alternative to CUSTOM + STEP parallel imaging acceleration. To test the performance of SENSE acceleration, SENSE-accelerated scans were compared to CUSTOM undersampled scans in the same six volunteers (age 28–66 years, mean age 41 years, 1 male, and 5 female) as the full-sample comparison. No gadolinium contrast was administered. Scan times were adjusted to be similar between both scans (SENSE scan times were PDw VISTA-5:44 min, 2.25X SENSE acceleration; T1w VISTA-5:03 min, 2X SENSE acceleration; SNAP-5:47 min, 2X SENSE acceleration). Other imaging parameters (Table 1) were identical between the scans. The CUSTOM undersampled images were STEP reconstructed offline and image quality was compared by a neuroradiologist as described below.

### Evaluation of STEP reconstruction performance

CUSTOM undersampled acquisitions can be reconstructed natively on the scanner by zero-filled image reconstruction, whereas STEP currently requires offline processing. To test the additional improvement that STEP can provide, STEP was compared to zero-filled image reconstruction offline as follows. CUSTOM undersampled 3D-WALLI protocol was scanned on six volunteers (age 23–46 years, mean age 38 years, 1 male, and 5 female) without contrast injection: Survey scan was followed by 3D TOF, T1w VISTA, PDw DANTE VISTA, and SNAP. In two additional patients (90 and 58 years; 2 male) with known ICAD stenosis, a post-contrast T1w VISTA was also obtained after single-dose gadolinium contrast (off-label use) injection (0.1 mmol/kg of Prohance at 2 cc/s followed by bolus of equal volume of saline), and 3-min post-injection. Protocol parameters were optimized, such that total scan time is 30 min with all scans being 0.5-mm isotropic resolution. The CUSTOM undersampled images were then reconstructed offline into two sets to assess STEP reconstruction: (1) unsampled k-space locations were zero filled and then reconstructed by inverse Fourier transform, and (2) reconstructed using STEP. STEP reconstruction times were recorded for each sequence and the average reconstruction time for each sequence was calculated.

### Image rating

Three sets of comparisons were performed: CUSTOM + STEP vs fully sampled, CUSTOM + STEP vs SENSE, CUSTOM + STEP vs CUSTOM + Zero-fill Fourier transform. For each comparison, images from both sets were de-identified with respect to reconstruction method and patient information and compared side-by-side for all contrast weightings by an experienced board-certified neuroradiologist with 7 years of intracranial vessel wall rating experience. The three comparisons were performed independently in separate reading sessions. Wall depiction, blood suppression quality, image quality, and CSF suppression quality were evaluated on a three- to five-point scale (Table 2). The following arterial segments in each subject were assessed: right and left cavernous, supraclinoid and terminal internal carotid artery, and M1 and M2/3 segments of the middle cerebral artery, A1 and A2/3 segments of the anterior cerebral artery, P1 and P2/3 segments of the posterior cerebral artery, and the basilar artery. Thus, in each subject, 19 arterial segments were assessed.

**Table 2:** Rating scale for assessment of STEP reconstruction performance

Feature assessed	Scale
Image quality	1: Non-diagnostic
	2: Limited due to low SNR
	3: Limited due to image blurring
	4: Adequate
	5: Optimal
Wall depiction	1: Poorly visualized
	2: Visualized but blurry
	3: Well depicted
CSF suppression	1: No CSF suppression
	2: Partial CSF suppression
	3: Sufficient CSF suppression for outer wall evaluation
	4: Complete CSF suppression
Blood suppression	1: Bright lumen
	2: Inhomogeneous suppression
	3: Complete suppression

### Quantitative comparison of CUSTOM + STEP to fully sampled protocol

Matched locations were identified on fully sampled sequences and CUSTOM + STEP images from the six volunteers (age 23–46 years, mean age 38 years, 1 male, and 5 female). Lumen and outer wall contours were drawn on three consecutive locations of the basilar artery. The lumen signal (S_L_), wall signal (S_W_), lumen area, and wall area were measured. The signal of the CSF adjacent to wall was also measured. For SNAP, only the lumen was contoured and S_L_ was measured. The standard deviation of noise (*σ*_n_) was measured from a region-of-interest placed in the air region outside the head. Signal-to-noise ratio (SNR) of lumen (SNR_L_) and wall (SNR_W_) were calculated as their respective signals divided by *σ*_n_. Wall − lumen CNR was calculated as SNR_W_–SNR_L_. Wall–CSF CNR was also calculated in a similar manner. The ratio of wall-to-lumen signal was also calculated. Image sharpness measurements in the same image locations were calculated using two different measurement methods as blur [14] and perceptual image sharpness (PSI) [15]. Lower blur values are better, whereas larger PSI values are better.

### Statistical analysis

Image ratings were summarized as mean ± standard deviation (SD). Ratings were aggregated by subject by averaging the ratings across all vessel segments. Average image ratings were compared between different techniques (fully sampled, SENSE, CUSTOM + zero fill, and CUSTOM + STEP) using the Wilcoxon signed-rank test. Image ratings of the techniques were also compared graphically for each vessel segment individually to assess the patterns of improvements in image quality across segments. The left and right sides were averaged and plotted as a bar chart. Quantitative measurements (lumen area, wall area, SNR, CNR, signal ratios, and sharpness metrics) were compared between fully sampled and CUSTOM + STEP images using the paired *t* test. The intraclass correlation coefficient (ICC) was used to summarize overall agreement in lumen and wall measurements made from fully sampled and CUSTOM + STEP images. All statistical calculations were conducted with the statistical computing language R (version 3.1.1; R Foundation for Statistical Computing, Vienna, Austria). Throughout, two-sided tests were used, with statistical significance defined as *p* < 0.05.

## Results

### Comparison to fully sampled protocol

**Fig. 2:**
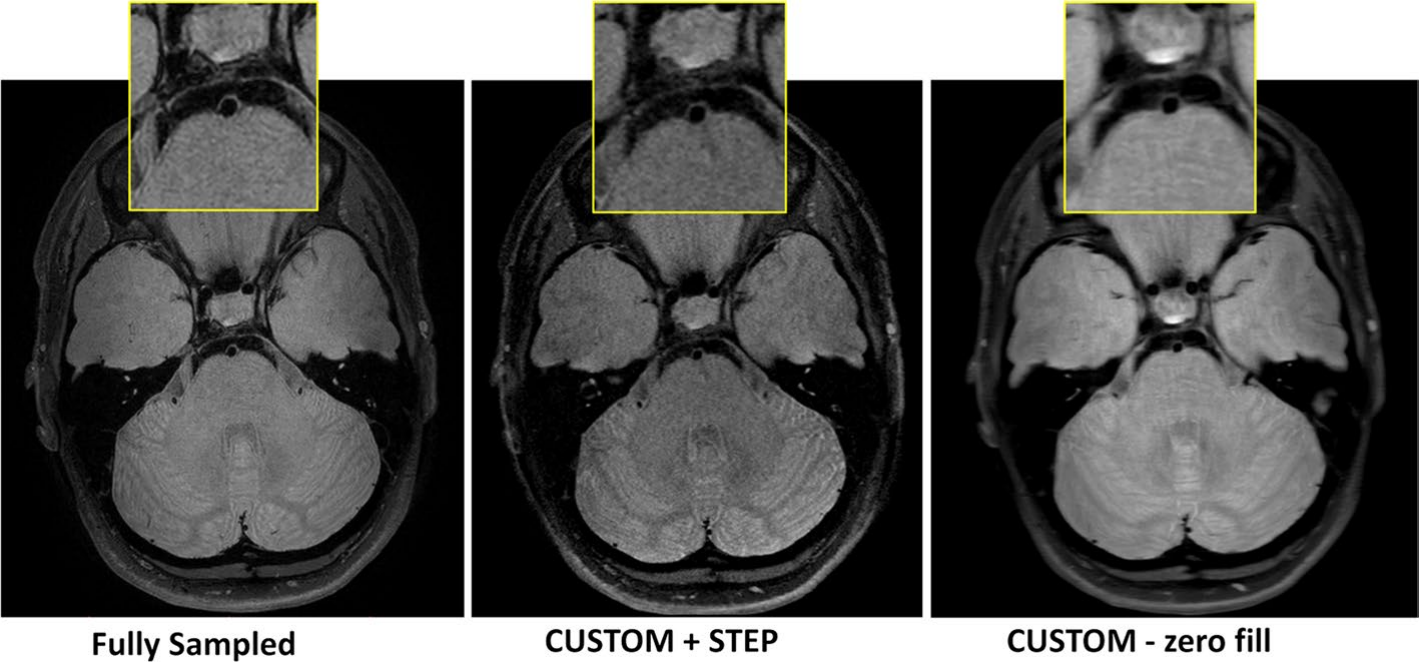
CUSTOM + STEP PDw VISTA compared to fully sampled PDw VISTA. Insets show the delineation of basilar artery wall. CUSTOM + STEP PDw VISTA can be seen to have similar vessel wall delineation as fully sampled PDw VISTA. CUSTOM + STEP provides improved vessel wall delineation than zero-filled Fourier reconstruction (right column), showing that both CUSTOM and STEP are required for accelerated IVW

**Fig. 3:**
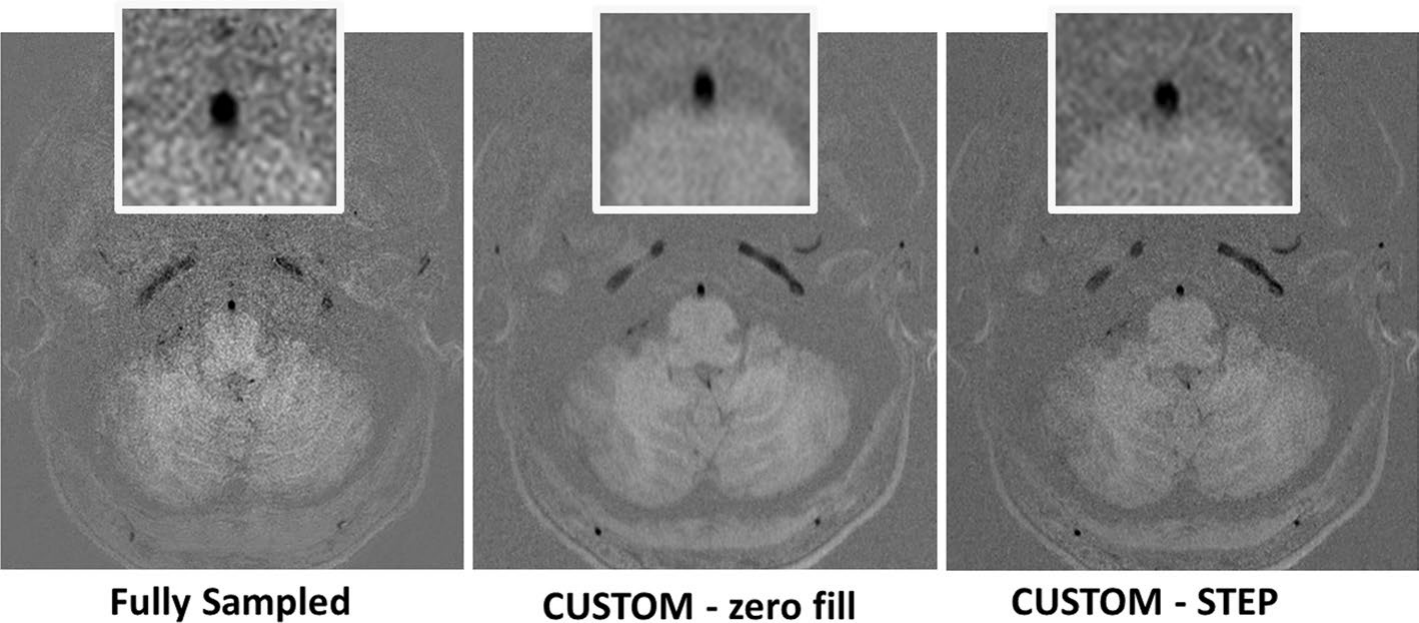
Phase-corrected real image of SNAP used to detect intraplaque hemorrhage and identify luminal boundaries as MRA is shown. CUSTOM + STEP SNAP can be seen to provide the same basilar artery lumen boundary as fully sampled SNAP. While fully sampled SNAP appears to have the best sharpness, CUSTOM + STEP SNAP has lower noise and improved brain tissue boundaries. SNAP with CUSTOM undersampling but with zero-filled Fourier reconstruction (middle column) provides adequate signal but blurred luminal boundaries compared to STEP reconstruction. This comparison shows the advantage of CUSTOM undersampling in enabling higher SNR acquisition for SNAP and subsequent joint STEP reconstruction

**Fig. 4:**
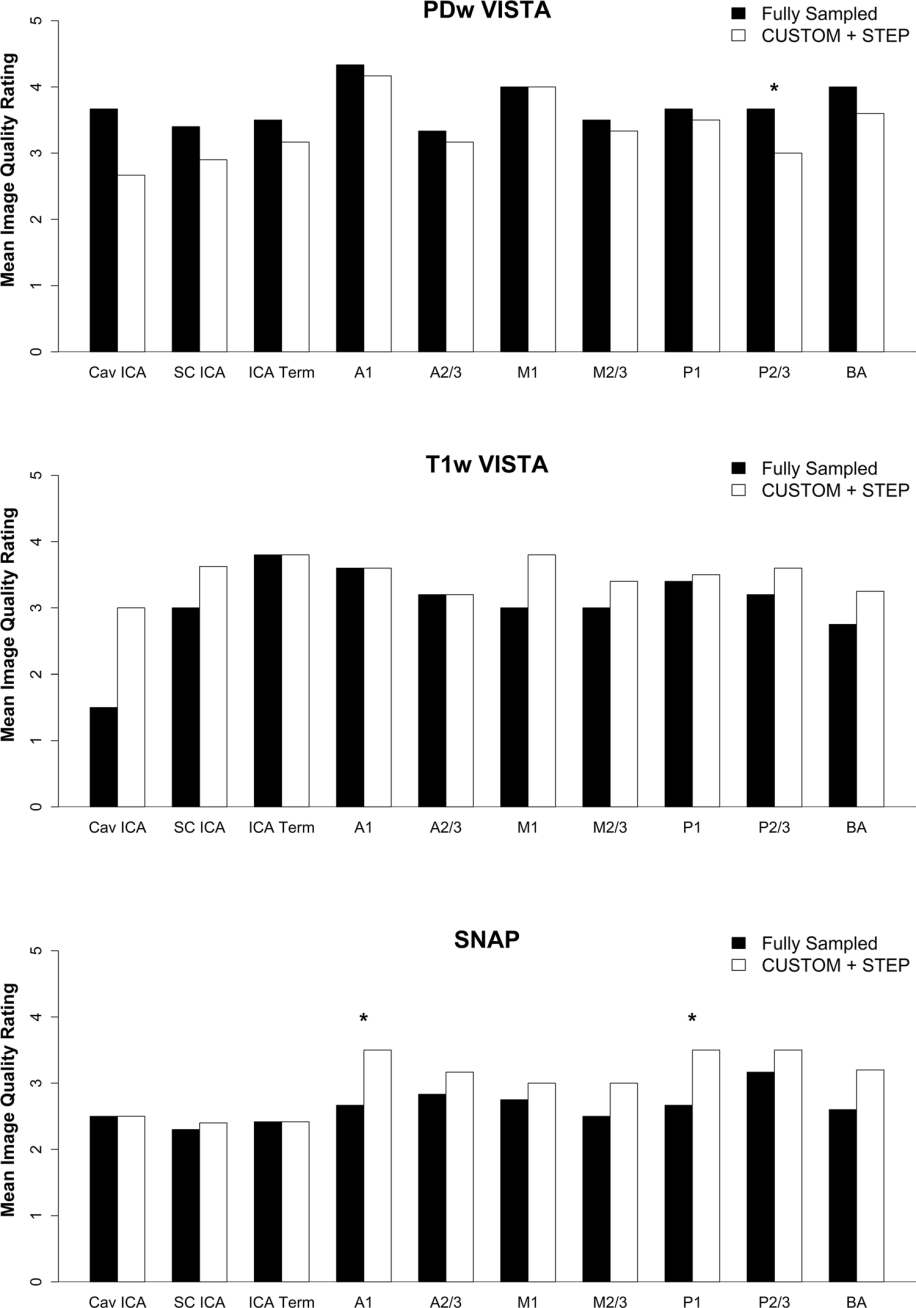
Bar chart of fully sampled vs CUSTOM + STEP image quality rating for each vessel (mean of left and right side). *A1* first segment of ACA, *A2/3* second and third segment of ACA, *ACA* anterior cerebral artery, *BA* basilar artery, *Cav ICA* cavernous ICA, *ICA* internal carotid artery, *ICA Term* ICA terminus, *M1* first segment of the MCA, *M2/3* second and third segments of the MCA, *MCA* middle cerebral artery, *P1* first segment of the PCA, *P2/3* second and third segments of the PCA, *PCA* posterior cerebral artery, *SC ICA* supraclinoid ICA

CUSTOM undersampled PDw VISTA and T1 VISTA reconstructed with STEP provided comparable vessel wall delineation (Fig. 2, Table 3) as fully sampled counterparts. CUSTOM + STEP SNAP showed similar luminal contrast as fully sampled SNAP (Fig. 3). Image quality ratings by the neuroradiologist did not differ significantly for T1w VISTA (mean 3.6 vs. 3.2, *p* = 0.62), were better for CUSTOM + STEP SNAP (mean 3.0 vs. 2.7, *p* = 0.031), and were lower for CUSTOM + STEP PDw VISTA (mean 3.4 vs. 3.7, *p* = 0.031) than fully sampled versions. Image quality rating was numerically higher across nearly all segments for T1w- and SNAP for CUSTOM + STEP but lower for PDw VISTA (Fig. 4). There were no significant differences in wall depiction or blood suppression for any sequence. CSF suppression rating was lower for CUSTOM + STEP on PDw VISTA (mean 2.7 vs. 3.0, *p* = 0.031) but similar for T1w VISTA (mean 2.9 vs. 2.8, *p* > 0.99).

**Table 3:** Comparison of image quality metrics (all segments averaged)

Metric	PDw VISTA			T1w VISTA			SNAP		
	Technique		*p* Value^a^	Technique		*p* Value^a^	Technique		*p* Value^a^
	Full	CUSTOM + STEP		Full	CUSTOM + STEP		Full	CUSTOM + STEP	
Fully sampled vs CUSTOM + STEP comparison									
Image quality (1–5)	3.7 ± 0.4	3.4 ± 0.4	**0.031**	3.2 ± 1.0	3.6 ± 0.3	0.62	2.7 ± 0.5	3.0 ± 0.3	**0.031**
Wall depiction (1–3)	2.4 ± 0.2	2.3 ± 0.2	0.12	2.2 ± 0.5	2.3 ± 0.3	> 0.99	–	–	–
CSF suppression (1–4)	3.0 ± 0.2	2.7 ± 0.2	**0.031**	3.1 ± 0.3	3.0 ± 0.2	0.19	–	–	–
Blood suppression (1–3)	2.9 ± 0.1	2.8 ± 0.2	0.88	2.8 ± 0.3	2.9 ± 0.1	> 0.99	2.5 ± 0.4	2.6 ± 0.2	0.31

Metric	PDw VISTA			T1w VISTA			SNAP		
	Technique		*p* Value^a^	Technique		*p* Value^a^	Technique		*p* Value^a^
	SENSE	CUSTOM + STEP		SENSE	CUSTOM + STEP		SENSE	CUSTOM + STEP	
SENSE vs CUSTOM + STEP comparison									
Image quality (1–5)	3.0 ± 0.5	3.3 ± 0.2	0.19	2.7 ± 0.4	3.4 ± 0.1	**0.031**	2.0 ± 0.2	3.1 ± 0.4	**0.031**
Wall depiction (1–3)	1.9 ± 0.2	2.0 ± 0.2	0.62	1.8 ± 0.3	2.2 ± 0.3	**0.031**	–	–	–
CSF suppression (1–4)	3.0 ± 0.3	3.0 ± 0.2	0.75	3.3 ± 0.1	3.3 ± 0.1	0.56	–	–	–
Blood suppression (1–3)	2.8 ± 0.2	3.0 ± 0.0	0.19	2.9 ± 0.1	3.0 ± 0.1	0.12	2.4 ± 0.4	2.8 ± 0.3	**0.031**

Metric	PDw VISTA			T1w VISTA			SNAP		
	Technique		*p* Value^a^	Technique		*p* Value^a^	Technique		*p* Value^a^
	CUSTOM + zero fill	CUSTOM + STEP		CUSTOM + zero fill	CUSTOM + STEP		CUSTOM + zero fill	CUSTOM + STEP	
CUSTOM + Zero fill vs CUSTOM + STEP comparison									
Image quality (1–5)	3.2 ± 0.6	3.7 ± 0.5	**0.008**	3.2 ± 0.6	4.0 ± 0.4	**0.008**	3.5 ± 0.8	3.4 ± 0.8	0.25
Wall depiction (1–3)	2.1 ± 0.4	2.4 ± 0.3	**0.008**	2.3 ± 0.5	2.6 ± 0.3	**0.016**	–	–	–
CSF suppression (1–4)	2.9 ± 0.4	3.0 ± 0.3	0.50	3.6 ± 0.3	3.6 ± 0.2	0.50	–	–	–
Blood suppression (1–3)	2.9 ± 0.1	2.9 ± 0.1	> 0.99	2.9 ± 0.1	3.0 ± 0.0	> 0.99	2.7 ± 0.2	2.6 ± 0.3	0.50

Significant *p*-values shown in bold font

^a^Wilcoxon signed-rank test

### Comparison to SENSE parallel imaging acceleration

**Fig. 5:**
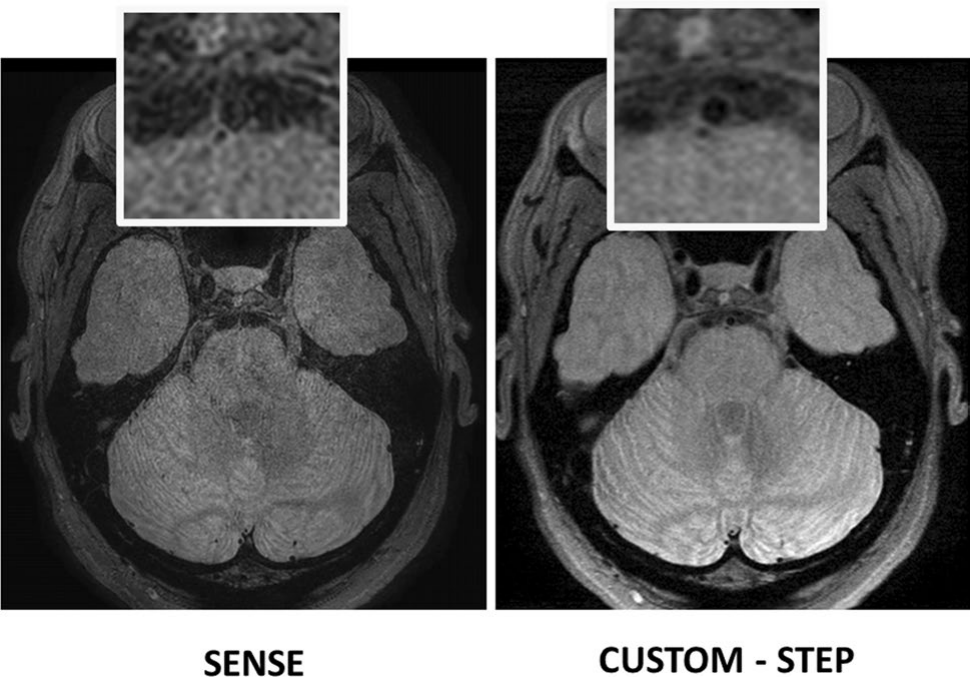
CUSTOM + STEP PDw VISTA shows superior performance to SENSE acceleration at the same scan time. Inset shows basilar artery can be clearly delineated by CUSTOM + STEP, but basilar artery wall is not visualized by SENSE acceleration. This comparison shows the advantage of using CUSTOM + STEP vessel wall-specific acceleration strategy

**Fig. 6:**
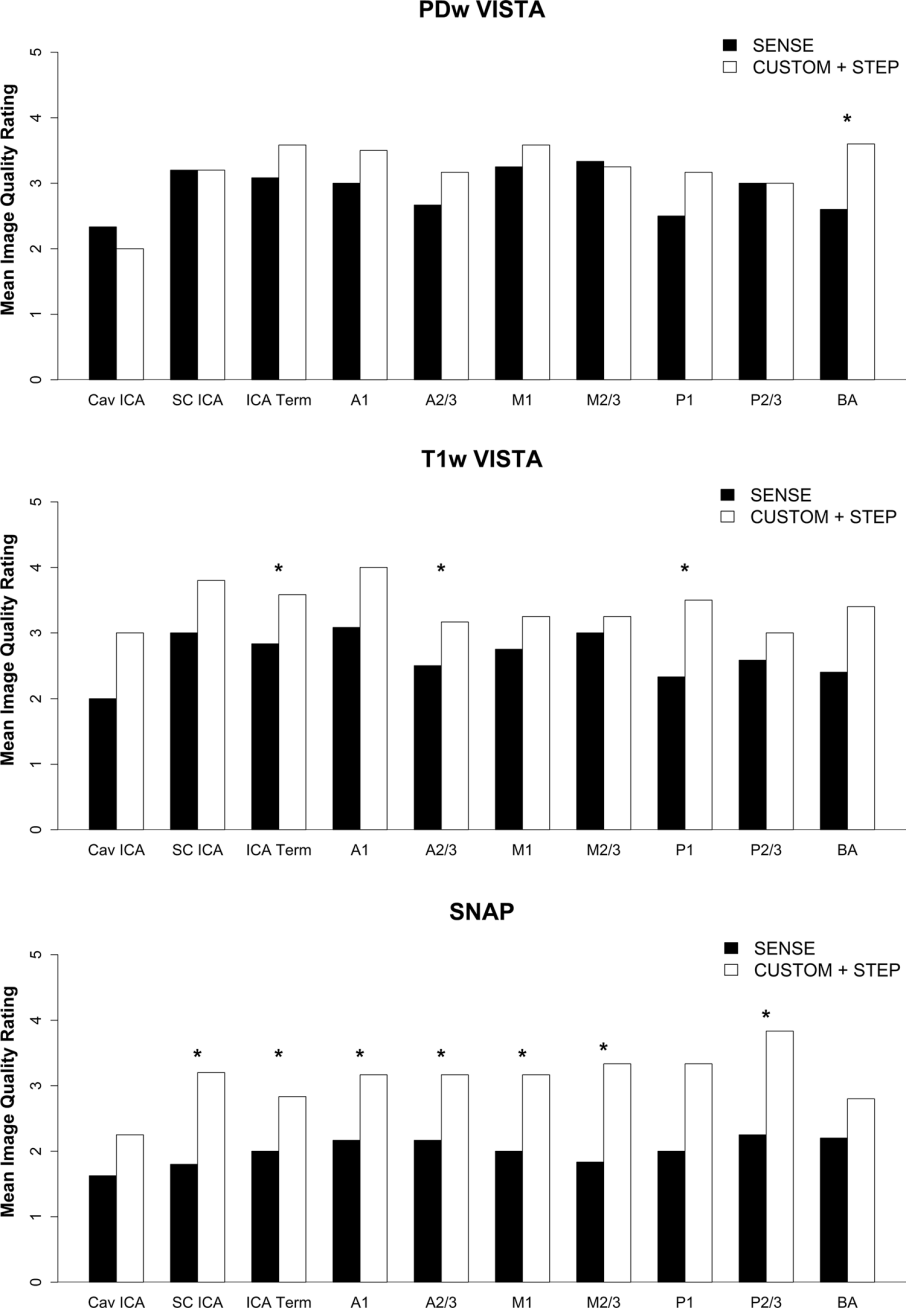
Bar chart of SENSE vs CUSTOM + STEP image quality rating for each vessel by reconstruction technique (mean of left and right side). *A1* first segment of ACA, *A2/3* second and third segments of ACA, *ACA* anterior cerebral artery, *BA* basilar artery, *Cav ICA* cavernous ICA, *ICA* internal carotid artery, *ICA Term* ICA terminus, *M1* first segment of the MCA, *M2/3* second and third segments of the MCA, *MCA* middle cerebral artery, *P1* first segment of the PCA, *P2/3* second and third segments of the PCA, *PCA* posterior cerebral artery, *SC ICA* supraclinoid ICA

**Fig. 7:**
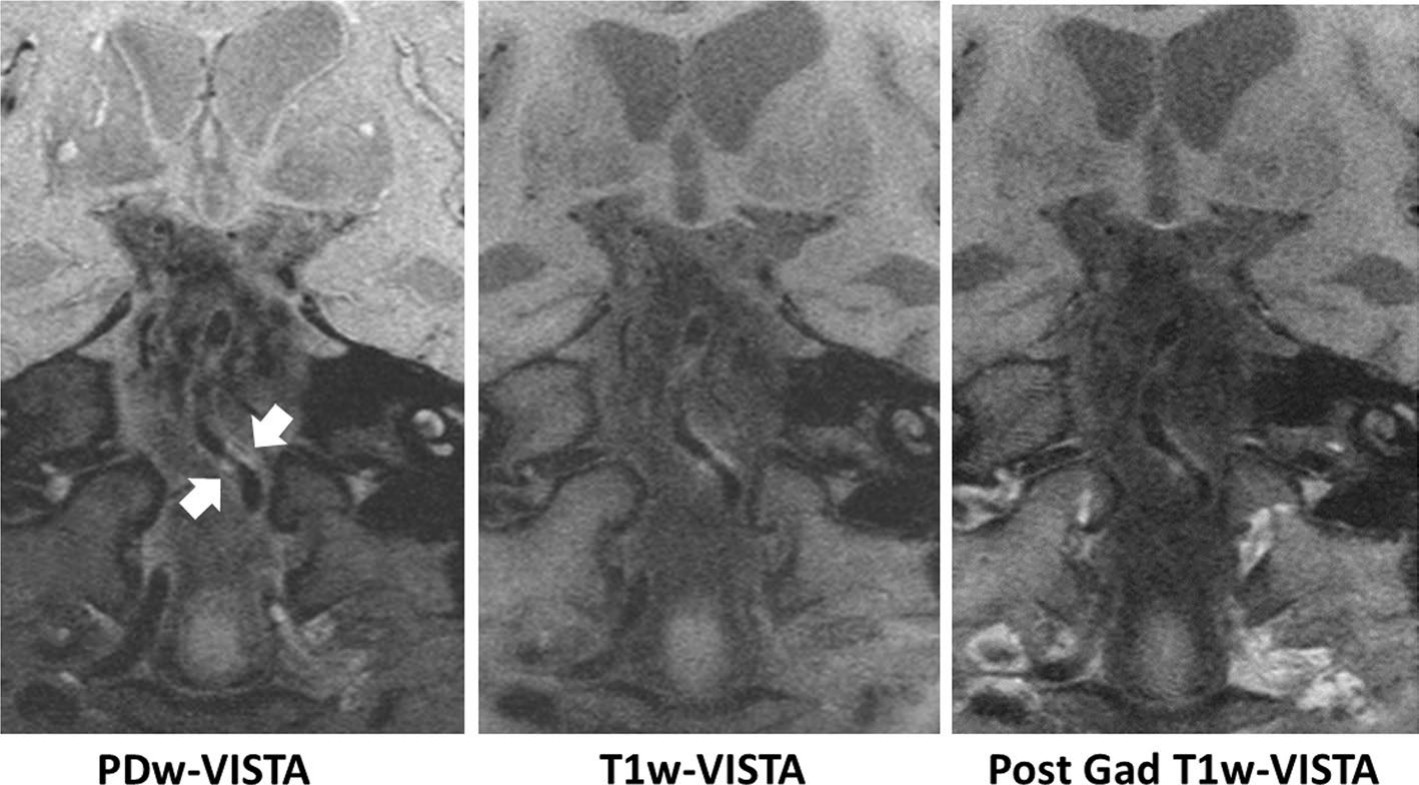
Basilar artery plaque visualized by accelerated ICAD IVW. Plaque (arrows) is seen on PDw VISTA, T1w VISTA, and post-contrast T1w VISTA

SENSE reconstructed SNAP and T1w VISTA had significantly lower image quality ratings than CUSTOM + STEP (*p* = 0.031 for both). CUSTOM + STEP T1w VISTA also showed better wall depiction (*p* = 0.031) than SENSE (Table 3). SENSE acceleration did not provide comparable SNR to CUSTOM + STEP for PDw VISTA as demonstrated in Fig. 5. There was no statistical difference in image quality on PDw VISTA (*p* = 0.19), although SENSE PDw VISTA tended to have a numerically lower image quality rating by the neuroradiologist across most segments (Fig. 6). There were also no significant differences in CSF suppression (*p* = 0.19). Blood suppression on SNAP was better using CUSTOM + STEP than SENSE (mean 2.8 vs. 2.4, *p* = 0.031, Table 3). Image quality was numerically higher across all segments for T1w VISTA and SNAP for CUSTOM + STEP when compared to SENSE (Fig. 7).

### Quantitative comparison of CUSTOM + STEP to fully sampled protocol

SNR and sharpness measurements are provided in Table 4. There was no significant difference in SNR_L_ between fully sampled and CUSTOM + STEP images for any sequence (*p* > 0.2 for each). SNR_W_ tended to be lower on CUSTOM + STEP than fully sampled on PDw DANTE VISTA (*p* = 0.062) and T1w VISTA (*p* = 0.019). Wall–lumen CNR was correspondingly lower for CUSTOM + STEP on both PDw and T1w VISTA (*p* < 0.033), but there were no significant differences in wall-to-lumen signal ratio for these sequences (*p* > 0.15 for all). The Wall–CSF CNR also tended to be lower for CUSTOM − STEP on PDw (*p* = 0.080) and T1w VISTA (*p* = 0.15) compared to fully sampled. Image blurring as indicated by the blur measure was lower for fully sampled on PDw VISTA (*p* < 0.001) and SNAP (*p* < 0.001) but not on T1w VISTA (*p* = 0.33), while perceptual image sharpness as indicated by the PSI measure tended to be better for CUSTOM + STEP on PDw VISTA (*p* < 0.001), T1w VISTA (*p* < 0.001), and SNAP (*p* = 0.069).

**Table 4:** Quantitative measurements of signal and noise

Variable	PDw VISTA					T1w VISTA					SNAP				
	Technique^a^		Difference^b^		*p* Value	Technique^a^		Difference^b^		*p* Value	Technique^a^		Difference^b^		*p* Value
	Full	CUSTOM − STEP	Mean	(95% CI)		Full	CUSTOM − STEP	Mean	(95% CI)		Full	CUSTOM − STEP	Mean	(95% CI)	
Lumen SNR	21.3 ± 6.9	17.2 ± 2.9	− 4.1	(− 11.1, 3.0)	0.20	14.8 ± 3.3	11.8 ± 8.1	− 3.0	(− 10.4, 4.4)	0.33	6.2 ± 2.5	5.7 ± 3.2	− 0.5	(− 1.7, 0.7)	0.33
Wall SNR	61.8 ± 18.1	46.9 ± 12.9	− 15.0	(− 31.0, 1.1)	0.062	36.2 ± 8.6	24.1 ± 10.1	− 12.1	(− 20.9, − 3.2)	**0.019**					
Wall–lumen CNR^c^	40.6 ± 12.4	29.7 ± 10.1	− 10.9	(− 20.4, − 1.3)	**0.033**	21.4 ± 7.1	12.3 ± 3.4	− 9.1	(− 14.9, − 3.3)	**0.012**	73.0 ± 80.0	22.4 ± 5.6	− 50.6	(− 131.8, 30.7)	0.17
Signal ratio^d^	3.0 ± 0.6	2.7 ± 0.3	− 0.3	(− 0.7, 0.1)	0.15	2.5 ± 0.6	2.3 ± 0.6	− 0.2	(− 1.0, 0.6)	0.51	6.2 ± 2.5	5.7 ± 3.2	− 0.5	(− 1.7, 0.7)	0.33
Brain SNR	92.0 ± 21.1	84.2 ± 16.2	− 7.8	(− 26.6, 10.9)	0.33	73.0 ± 10.4	54.7 ± 15.0	− 18.3	(− 33.9, − 2.7)	**0.031**					
Wall–CSF CNR	31.9 ± 17.4	23.5 ± 13.7	− 8.4	(− 18.2, 1.4)	0.080	9.4 ± 12.9	5.0 ± 8.4	− 4.4	(− 11.3, 2.5)	0.15					
Blur	0.29 ± 0.01	0.35 ± 0.01	0.06	(0.05, 0.06)	**< 0.001**	0.36 ± 0.01	0.36 ± 0.01	0.004	(− 0.006, 0.014)	0.33	0.17 ± 0.01	0.23 ± 0.01	0.06	(0.06, 0.07)	**< 0.001**
PSI	0.37 ± 0.01	0.45 ± 0.02	0.08	(0.06, 0.10)	**< 0.001**	0.37 ± 0.01	0.46 ± 0.01	0.09	(0.08, 0.10)	**< 0.001**	0.47 ± 0.01	0.48 ± 0.01	0.01	(− 0.00, 0.03)	0.069

Significant *p*-values shown in bold font

^a^Mean ± SD (mm^2^)

^b^CUSTOM − STEP minus fully sampled

^c^Wall–lumen CNR for PDw and T1w VISTA; noise–lumen CNR for SNAP

^d^Wall:lumen signal ratio for PDw and T1w VISTA; noise:lumen signal ratio for SNAP

Morphometric measurements are provided in Table 5. There was good agreement in lumen area measurements for each sequence (ICC 0.92–0.99) with no significant difference between fully sampled and CUSTOM − STEP (*p* > 0.27 for each). Similarly, agreement in wall area measurements was also good for PDw (ICC 0.90) and T1w VISTA (ICC 0.96) with no significant difference between fully sampled and CUSTOM − STEP (*p* > 0.10).

**Table 5:** Morphometric measurements

Sequence	Lumen area					Wall area				
	Technique^a^		*p* Value^b^	Agreement		Technique^a^		*p* Value^b^	Agreement	
	Full	CUSTOM − STEP		ICC	(95% CI)	Full	CUSTOM − STEP		ICC	(95% CI)
PDw VISTA	7.6 ± 3.9	7.7 ± 3.7	0.59	0.99	(0.96, 1.00)	8.4 ± 2.7	9.1 ± 2.0	0.10	0.90	(0.41, 0.99)
T1w VISTA	7.0 ± 3.4	6.8 ± 3.2	0.27	0.99	(0.96, 1.00)	7.7 ± 3.7	8.2 ± 3.0	0.25	0.96	(0.74, 1.00)
SNAP	6.2 ± 2.5	5.7 ± 3.2	0.33	0.92	(0.60, 0.99)					

Significant *p*-values shown in bold font

^a^Mean ± SD (mm^2^)

^b^Paired *t* test comparing fully sampled and CUSTOM − STEP measurements

### Evaluation of protocol performance

The full 3DWALLI protocol was well tolerated by patients. ICAD plaque was clearly visualized in patients (Fig. 7). PDw VISTA, T1w VISTA, and post-contrast T1w VISTA were reconstructed in less than 20 min per volume using STEP reconstruction. Joint STEP reconstruction of SNAP required about 70 min per volume (Table 6 shows average reconstruction times).

**Table 6:** STEP reconstruction time

	PDw VISTA	T1 VISTA	SNAP Slab 1	SNAP Slab 2	Post T1 VISTA
*N*	8	8	7	7	2
Reconstruction time (min)	19.08 ± 1.38	18.64 ± 1.83	70.92 ± 6.28	71.74 ± 4.80	19.87 ± 2.49

### Evaluation of STEP reconstruction performance

**Fig. 8:**
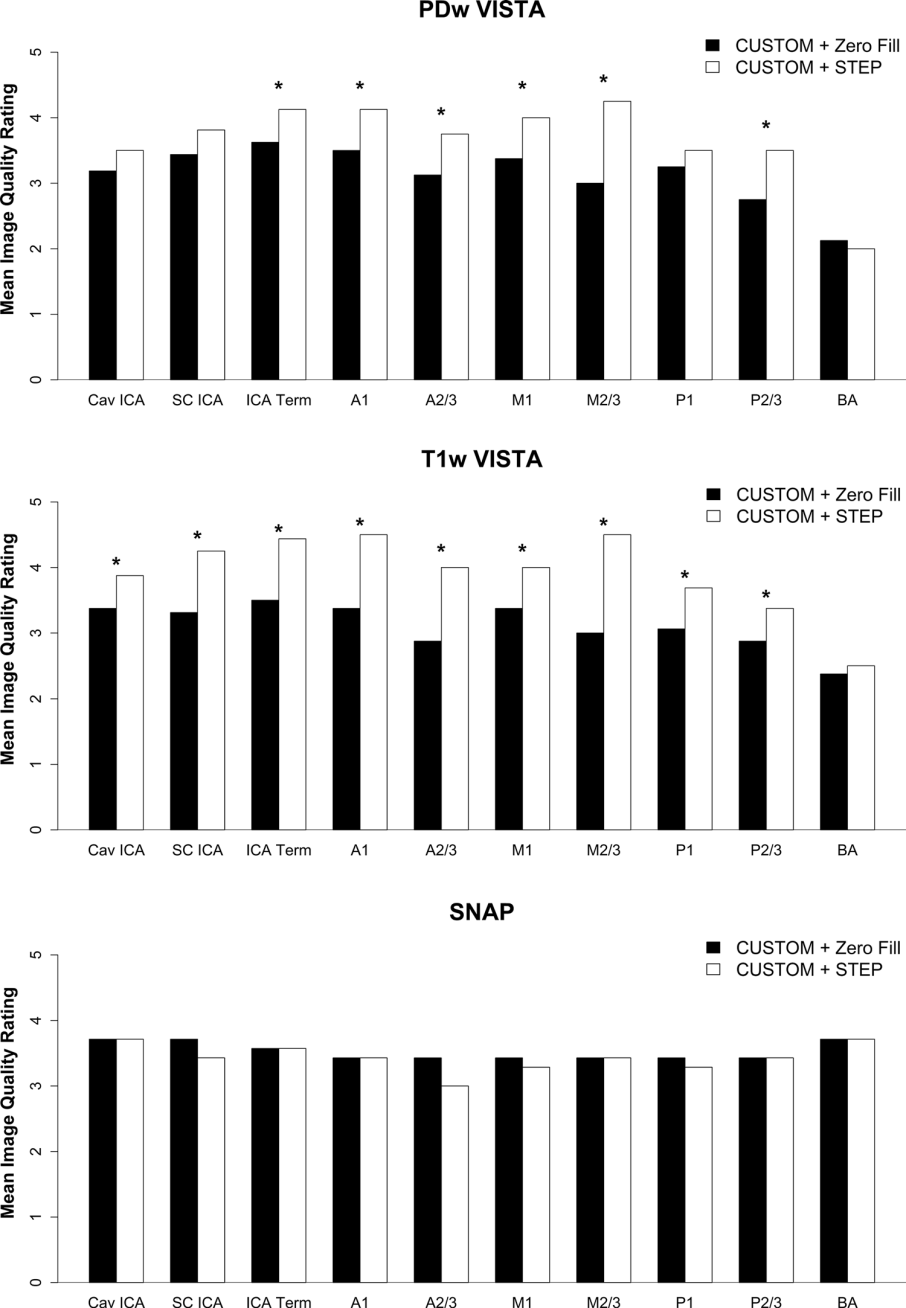
Bar chart of CUSTOM + zero-filled FFT vs CUSTOM_STEP image quality rating for each vessel by reconstruction technique (mean of left and right sides). *A1* first segment of ACA, A2/3 second and third segments of ACA, *ACA* anterior cerebral artery, *BA* basilar artery, *Cav ICA* cavernous ICA, *ICA* internal carotid artery, *ICA Term* ICA terminus, *M1* first segment of the MCA, *M2/3* second and third segments of the MCA, *MCA* middle cerebral artery, *P1* first segment of the PCA, *P2/3* second and third segments of the PCA, *PCA* posterior cerebral artery, *SC ICA* supraclinoid ICA

After acceleration by CUSTOM, STEP reconstruction provided improved image sharpness compared to zero-filled image reconstruction, as shown in Figs. 2 and 3. Average image quality and wall depiction ratings were significantly improved using STEP reconstruction for PDw VISTA (*p* < 0.008 for each) and T1w-VISTA (*p* ≤ 0.016 for each) (Table 3). Image quality was numerically higher across nearly all segments for PDw- and T1w-VISTA by STEP compared with zero-filled reconstruction (Fig. 8). There were no significant differences in CSF or blood suppression. For SNAP, no significant differences in image quality, CSF suppression, or blood suppression were seen.

## Discussion

We demonstrated a multi-contrast 3D 0.5-mm isotropic resolution IVW protocol (3D-WALLI) with a scan time of less than 30 min. 3D-WALLI consists of highly T1w SNAP, PDw DANTE prepared VISTA, T1w VISTA, and post-contrast T1w VISTA to enable comprehensive characterization of intracranial vasculopathies. The scan time of 5 min per sequence was achieved using CUSTOM k-space undersampling. The reduction in scan time per sequence and for the full 3D-WALLI protocol was designed to benefit high-resolution IVW by reducing the chances of motion blurring secondary to patient movement.

We employed CUSTOM k-space undersampling and STEP reconstruction methods for imaging acceleration while maintaining optimum vessel wall visualization. STEP is a partially parallel image reconstruction method that is tailored for magnetization prepared sequences such as vessel wall MRI sequences. STEP has been shown to provide improved vessel wall visualization [8] when compared to the other partially parallel imaging methods such as SPIRiT [9]. Compared to other k-space undersampling strategies such as variable density undersampling, CUSTOM is designed to provide consistently effective interpolation condition within local k-space regions for parallel imaging reconstruction while providing higher sampling density at the center of k-space for less aliasing artifact. Thus, CUSTOM provides adequate support for STEP reconstruction, and due to increased SNR, it is ideally suited for IVW.

IVW requires high spatial resolution, since the normal intracranial arterial wall is 0.2–0.3-mm-thick [6]. The previous studies have demonstrated the utility of high-resolution 2D cross-sectional IVW [16–18]. These studies acquired one to a few 2-mm-thick slices perpendicular to the axis of the MCA or basilar artery. Unlike arteries with relatively straight courses such as the common carotid artery or abdominal aorta, segments of the intracranial vessels are tortuous and the location where lesions may form is variable. Thus, 3D isotropic sequences are important for IVW, such that multi-planar reformatting in arbitrary planes allows torturous vessels to be observed in cross section. In addition, increased brain coverage with 3D IVW as compared to 2D IVW allows for the detection and evaluation of multiple lesions, including culprit lesions that may not be prospectively detected on luminal imaging due to outward remodeling/lack of stenosis [19, 20].

Recent studies have demonstrated 3D isotropic scans for IVW [12, 21–26]. A single contrast weighting was used in these studies to identify plaque with or without contrast injection. Two studies [27, 28] indicated the need for multi-contrast IVW and used three-to-four contrast weightings. In addition, multi-contrast protocols have shown value for intracranial vasculopathy differentiation [2, 5]. However, 3D IVW spatial resolutions of 0.6–0.8-mm isotropic and/or scan times per sequence of up to 12 min in those studies may be limiting for patients. In this study, both 0.5-mm isotropic resolution and 5–6-min scan times were targeted for broader applicability in IVW. 0.5-mm isotropic resolution is also recommended by the ASNR expert consensus document for IVW [6].

The value of specific contrast weightings as part of a multi-contrast IVW has been extensively investigated in extracranial carotid atherosclerotic plaques relative to histopathology. A multi-contrast protocol consisting of a highly T1w sequence, pre-contrast, and post-contrast T1w sequence and time-of-flight MRA are central for the assessment of plaque composition [29, 30] in extracranial carotid atherosclerotic disease. The requirements for IVW are less clear. We have chosen the contrast weightings in the current manuscript based on the recent consensus IVW document that suggests a protocol with TOF, pre-contrast T1, post-contrast T1, and T2 weightings. SNAP is a highly T1-weighted sequence used to identify intraplaque hemorrhage. SNAP also provides simultaneous angiography for stenosis assessment [13] in addition to 3D TOF. Pre-contrast and post-contrast T1w VISTA can be used to identify lipid core, loose matrix tissue and calcification, and to measure wall thickness. The proposed IVW protocol also acquires PDw VISTA as an intermediate contrast weighting instead of T2w VISTA. Use of DANTE prepulse with PDw VISTA allows complete suppression of cerebrospinal fluid required for identification of vessel wall. The normal vessel wall is difficult to distinguish on T2w VISTA due to bright CSF. T2w VISTA can, however, be substituted for PDw VISTA. CSF suppression ratings on CUSTOM + STEP were lower than fully sampled for the PDw VISTA comparison only and not on other comparisons. However, wall–CSF CNR measurements tended to be lower on CUSTOM + STEP for both PDw VISTA and T1w VISTA. The higher wall–CSF CNR measurement for fully sampled sequences was most likely due to the higher SNR and the perceived high brain tissue to CSF contrast likely led to higher CSF suppression ratings for fully sampled PDw VISTA.

A single fully sampled 0.5-mm isotropic scan in our study averaged 10 min for the T1 and PD contrast weightings which agrees with the other studies [23] with 0.56 mm isotropic resolution. Highly T1-weighted SNAP sequence at 0.5 mm requires multiple averages without CUSTOM to achieve sufficient image quality. Thus, the fully sampled 3D-WALLI protocol will require up to an hour without CUSTOM. Undersampling with CUSTOM provided two advantages with respect to protocol scan time. First, it allowed total scan time to be reduced to 30 min, even though two averages were acquired for all sequences. Second, the denser sampling of k-space center and averaging allowed for 0.5-mm isotropic SNAP MRI with joint STEP reconstruction. Joint STEP reconstruction was implemented in this work to utilize the higher SNR of SNAP reference image to improve reconstruction results. Thus, CUSTOM + STEP allowed 0.5-mm isotropic scans to be accelerated while improving image quality over fully sampled 0.5-mm isotropic scans. Reducing the scan time per sequence to 5 min and reducing the total protocol scan time are also expected to improve vessel wall visualization in patient studies through reduction in motion blurring secondary to patient movement.

Parallel imaging is clinically available on modern scanners for scan acceleration. We compared CUSTOM + STEP to SENSE for use in IVW. SENSE-accelerated 3D volumes had more severe SENSE fold-over artifacts, such that several images were not usable. In SNR constrained sequences such as T1w VISTA or SNAP, CUSTOM + STEP had higher image quality ratings compared to SENSE. Another option for scan acceleration is compressed sensing [31]. Compressed sensing is currently not available clinically and requires an undersampling and reconstruction strategy similar to CUSTOM + STEP. Variable density sampling and an L1 norm reconstruction with a wavelet domain transformation is customarily used for compressed sensing [31]. We note that CUSTOM undersampling is designed to support both parallel imaging and compressed sensing [7], and has been shown to reduce reconstruction error when compared to variable density sampling. STEP can also be combined with a wavelet basis and L1 norm minimization, and has been previously compared favorably against L1 SPIRiT [8].

Performance of CUSTOM + STEP was also compared to fully sampled acquisition. Image quality ratings confirmed similar performance especially for T1 VISTA and SNAP. While CNR was lower for CUSTOM + STEP than fully sampled, both subjective image quality rating and morphometric measurements were comparable. The reduction in CNR can be attributed to lower signal due to undersampling as shown by the reduced SNR_W_ on CUSTOM + STEP but comparable SNR_L_ on all sequences. However, the wall-to-lumen signal ratio and lumen area measurements on CUSTOM − STEP were comparable to fully sampled sequences, suggesting that vessel delineation is not impacted by the lower SNR. Perceptual image sharpness as shown by the PSI measure was better for CUSTOM + STEP despite the reduced blur measure. Wall area measurements were comparable to fully sampled sequences, suggesting that image sharpness required for morphometric measurements was sufficient.

Our study has the following limitations and scope for improvement. (1) Reconstruction was performed offline and such reconstructions are not clinically feasible considering workflow and volume of studies interpreted in most private and academic radiology practices. Performance of STEP reconstruction of IVW scans has not been previously demonstrated. This study shows that STEP can provide high-quality reconstruction of IVW and, therefore, justifies future efforts aimed at implementing online STEP reconstruction. (2) STEP reconstruction currently requires 20–70 min per sequence. Reconstruction times will need further increased efficiency if online reconstruction is to be implemented. GPU processors with additional cores are becoming increasingly available and may help to further reduce reconstruction times. (3) This technical development study compared CUSTOM + STEP image quality to fully sampled and SENSE-accelerated scans, and the optimized protocol was demonstrated using a limited number of subjects. While the comparison showed CUSTOM + STEP provides similar vessel wall delineation as fully sampled IVW and can be applied to patient studies, further evaluation in a wide range of ICAD lesions including large intracranial atherosclerotic plaques with multiple plaque components may be required to assess subtle differences between the imaging techniques. (4) The order of sequence acquisition was not random between CUSTOM undersampled and fully sampled scans. Thus, the effect of motion could not be ruled out when comparing the sequences. Future studies aimed at studying the effect of motion on high-resolution scans will be required to specifically study this effect.

## Conclusions

CUSTOM + STEP acceleration allows acquisition of a multi-contrast 3D 0.5-mm isotropic IVW protocol within 30 min of scan time. CUSTOM + STEP is superior to SENSE for IVW. STEP reconstruction of CUSTOM undersampled IVW provides improved vessel wall delineation compared to zero-filled image reconstruction. The CUSTOM + STEP protocol is well tolerated by patients and shows good delineation of ICAD plaque. Although some quantitative and qualitative scores for CUSTOM − STEP were lower than fully sampled IVW, CUSTOM + STEP provides comparable vessel wall delineation as fully sampled IVW. Combined with the fact that CUSTOM + STEP sequences are much shorter, the CUSTOM − STEP may provide a clinically feasible alternative to fully sampled IVW.

## Abbreviations

IVW: Intracranial vessel wall MRI
SNR: Signal-to-noise ratio
CUSTOM: Cartesian undersampling with target ordering method
STEP: Self-supporting tailored k-space estimation for parallel imaging reconstruction
3D WALLI: 3D intracranial wall imaging protocol
VISTA: Volume isotropic turbo spin echo acquisition
DANTE: Delay alternating with nutation for tailored excitation
SNAP: Simultaneous non-contrast angiography and intraplaque hemorrhage imaging
